# A High Visceral-To-Subcutaneous Fat Ratio is an Independent Predictor of Surgical Site Infection after Gastrectomy

**DOI:** 10.3390/jcm8040494

**Published:** 2019-04-11

**Authors:** Jung Ho Kim, Jinnam Kim, Woon Ji Lee, Hye Seong, Heun Choi, Jin Young Ahn, Su Jin Jeong, Nam Su Ku, Taeil Son, Hyoung-Il Kim, Sang Hoon Han, Jun Yong Choi, Joon-Sup Yeom, Woo Jin Hyung, Young Goo Song, Sung Hoon Noh

**Affiliations:** 1 Department of Internal Medicine, Yonsei University College of Medicine, Seoul 03722, Korea; qetu1111@yuhs.ac (J.H.K.); jam764@yuhs.ac (J.K.); leewj86@yuhs.ac (W.J.L.); shininghye@yuhs.ac (H.S.); bh138@yuhs.ac (H.C.); comebacktosea@yuhs.ac (J.Y.A.); jsj@yuhs.ac (S.J.J.); shhan74@yuhs.ac (S.H.H.); seran@yuhs.ac (J.Y.C.); joonsup.yeom@yuhs.ac (J.-S.Y.); imfell@yuhs.ac (Y.G.S.); 2 Department of Surgery, Yonsei University College of Medicine, Seoul 03722, Korea; cairus@yuhs.ac (H.-I.K.); wjhyung@yuhs.ac (W.J.H.); sunghoonn@yuhs.ac (S.H.N.)

**Keywords:** surgical site infection, gastric cancer, gastrectomy, body composition, visceral-to-subcutaneous fat ratio, risk factors, outcomes

## Abstract

Recent studies have shown that body composition is an important factor that affects surgical site infection (SSI). However, each study has utilized different body composition criteria. Therefore, in this study, we aim to determine the most predictable body composition criteria for the prediction of SSI after gastrectomy. The visceral fat area (VFA), subcutaneous fat area (SFA), and muscle area were assessed by a preoperative-stage computed tomographic (CT) scan. To compare the predictive performance of body composition for SSI, logistic regression models were used, and the models were compared using the receiver operation characteristic (ROC) curve and the area under the curve (AUC) value. Of the 1038 eligible patients, 58 patients (5.6%) developed SSI. The VFA-to-SFA ratio showed the best predictive performance (mean AUC 75.11). The cutoff value for the SSI of the VFA-to-SFA ratio was 0.94, and the sensitivity and specificity were 67.86% and 77.65%, respectively. A multivariate logistic analysis indicated that a total gastrectomy (OR, 2.13; *p* = 0.017), stage III or IV cancer (OR, 2.66; *p* = 0.003), and a high VFA-to-SFA ratio (OR, 8.09; *p* < 0.001) were independent risk factors for SSI after gastrectomy. The VFA-to-SFA ratio is the most predictable body composition model for use in predicting the incidence of SSI after gastrectomy.

## 1. Introduction

Gastric cancer is the fifth most common cancer in the world and the third most common cause of cancer-related death [1]. Surgery is the main treatment for gastric cancer, and this treatment is one of the most common surgeries performed in South Korea [2,3]. Although improvements to surgical devices and advances in surgical techniques have resulted in reduced mortality after gastrectomy, postoperative complications remain a clinically significant problem [4]. 

Surgical site infection (SSI) is one of the most common postoperative complications of gastrectomy. SSI is associated with prolonged hospitalization and increased mortality [5]. Therefore, it is important to investigate the risk factors for SSI.

Recently, the effects of different body compositions on SSI have been attracting attention. Especially, the effects of fat distribution and loss of skeletal muscle, called sarcopenia, have been focused on. Some studies have shown that visceral fat is a risk factor for postoperative complications in digestive surgery [6,7,8]. Sarcopenia, is also known to be associated with complications following digestive surgery [9,10,11]. However, each of these studies has used different body composition criteria. Because there are differences in body composition between patients from different ethnic backgrounds and regions, it is difficult to define the body composition criteria based on a specific value. To our knowledge, universally applicable criteria to define body composition have yet to be established. In addition, as almost all patients are given a computed tomographic (CT) scan before gastric cancer surgery, measuring body composition using a preoperative CT scan for predicting SSI could be worthwhile. Therefore, in this study, we aimed to investigate the most predictable body composition criteria to predict SSI after gastrectomy using a preoperative CT scan.

## 2. Materials and Methods

### 2.1. Study Design and Population

**Figure 1:**
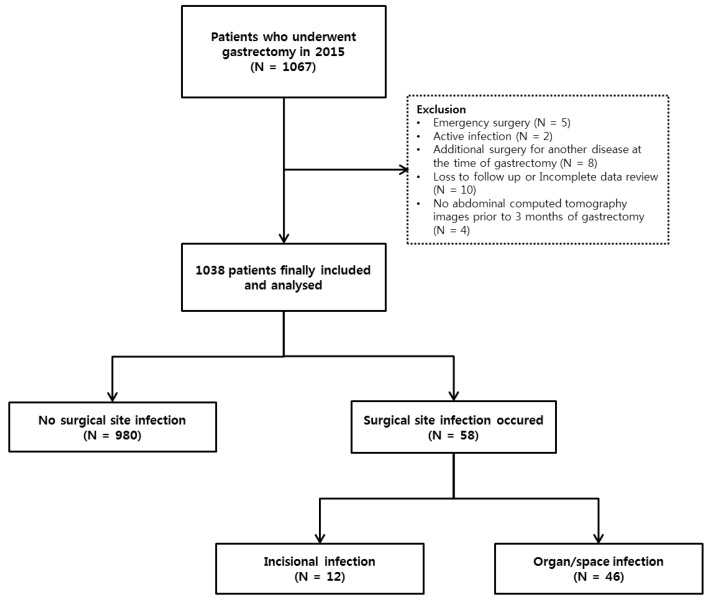
Flow diagram of study process.

Between January 2015 and December 2015, 1067 patients underwent gastrectomy for gastric cancer in the Severance Hospital in Seoul, Korea. We the excluded patients who required emergency surgery, those with active infections, and those for whom a preoperative CT scan was unavailable. Consequently, we enrolled 1038 patients who underwent gastrectomy and who did not qualify for exclusion. A schematic flow of the study is shown in Figure 1. The study was approved by the Institutional Review Board (IRB) of Yonsei University Health System Clinical Trial Center (4-2017-1252). Because the study was retrospective and the study subjects were anonymized, the IRB waived the requirement for written consent from the patients.

### 2.2. Measurement of Visceral Fat Area, Subcutaneous Fat Area, and Total Abdominal Muscle Area

**Figure 2:**
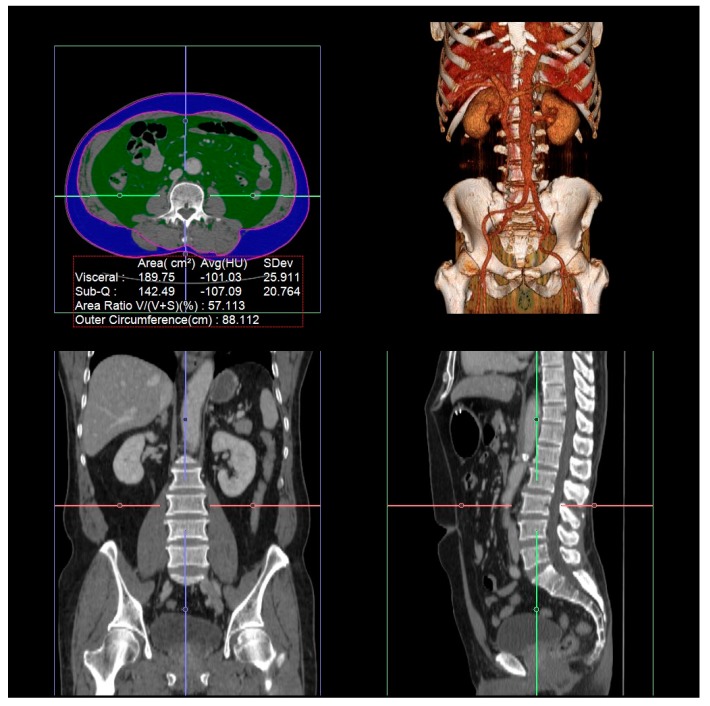
Example of body composition measurement at the level of the third lumbar vertebra on a computed tomography scan in the Hounsfield unit by AquariusNET Server.

The visceral fat area (VFA), subcutaneous fat area (SFA), and total abdominal muscle area (TAMA) of the patients were assessed using the latest preoperative CT scans. The cross-sectional VFA, SFA, and TAMA values were measured at the level of the third lumbar vertebra, as previously described [12]. Specific tissue distinction was performed by image analysis software based on Hounsfield unit (HU) thresholds, using an AquariusNET Server (TeraRecon, Foster City, CA, USA). By summing the tissue pixels and multiplying by the pixel area, the cross-sectional areas were calculated automatically and the tissue boundaries were corrected manually when needed. The VFA and SFA were identified by adipose tissue thresholds of −150 to −50 HU and −190 to −30 HU, respectively [13]. The TAMA values for the paraspinal and abdominal wall muscles were calculated and quantified by thresholds of −29 to 150 HU [14]. The cross-sectional VFA and TAMA values were normalized for the patients’ heights and were reported in units of cm^2^/m^2^ as the visceral fat index and muscle index, respectively. Examples of the areas of VFA and SFA are depicted in Figure 2.

### 2.3. Definition of SSI

Surgeons checked for SSI every day during the patients' in-hospital stays, and at every outpatient clinic or emergency room visit until 30 days after surgery [15]. Patients usually visited the outpatient clinic around seven days after discharge. If the patient did not visit a scheduled outpatient appointment, the surgical nurse called to check for complications, including SSI, and contacted them to visit the clinic. SSI was defined based on the 1999 Centers for Disease Control and Prevention National Nosocomial Infection Surveillance System manual [16]. 

### 2.4. Surgical Procedures and Perioperative Management

Typical surgical procedures for open, laparoscopic, and robotic gastrectomy have been previously described in detail [17,18,19,20]. The da Vinci System (Intuitive Surgical, Sunnyvale, CA, USA) was used to perform robotic surgeries. Depending on the location of the tumors, a total or partial gastrectomy was performed. Lymph node dissection D1, D1+, or D2 was performed according to the Japanese gastric cancer treatment guidelines of 2014 [21]. The reconstruction type and method were selected according to the surgeons’ preferences [22]. 

### 2.5. Statistical Analysis

To verify the normality of the continuous variables, the Kolmogorov–Smirnov test and Shapiro–Wilk test were conducted. The independent t-test and the Mann–Whitney test were used to compare the continuous variables of the two groups. The Chi-square test or the Fisher exact test was used to compare the categorical variables. Among variables with *p* < 0.05 in the univariate analyses, we selected variables for multivariate logistic regression based on the clinical significance known by previous studies [23,24,25,26]. After checking multicollinearity, a multivariate logistic regression including an interaction term was performed to identify the independent risk factors for SSI. The interaction between gender and VFA-to-SFA ratio was verified at a *p*-value of 0.004. In order to compare the predictive performance of body composition for the SSI, logistic regression models were used, and the models were compared using the receiver operation characteristic (ROC) curve and the area under the curve (AUC) value. The AUC value was extracted by using the standard error estimated from the bootstrap samples. As a method of comparing the predictive performance of the models, we used the bootstrap samples to show the confidence interval for the difference, and we used the DeLong method to compare with the control. The consistency between the predicted and the observed probabilities of the actual data was assessed using the Hosmer–Lemeshow test. The cutoff value, the AUC value, and the sensitivity and specificity of the selected model were determined using the Youden method, and the odds ratio (OR) was obtained by multivariate logistic regression analysis. *p*-values of <0.05 were considered statistically significant. All of the statistical analyses were performed using the R package, version 3.4.4 (The R Foundation for Statistical Computing, Vienna, Austria).

## 3. Results

### 3.1. Clinical Characteristics of Patients with SSI after Gastrectomy

The clinical and surgical characteristics of the 1038 patients who had undergone gastrectomy are shown in Table 1. Among the 1038 participants, SSI occurred in 58 patients. Of these, 12 patients (20.7%) were diagnosed through post-discharge surveillance (incisional SSI for four patients (4/12, 33.3%); organ/space SSI for eight patients (8/46; 17.4%)). Male sex, smoking, an American Society of Anesthesiologists (ASA) score ≥3, neoadjuvant chemotherapy, and stage III or IV cancer were statistically significantly associated with SSI. For the variables associated with surgery, total gastrectomy, open surgery, D2 or more lymph node dissection, and longer operation times were significantly associated with SSI.

### 3.2. Risk Factors for SSI after Gastrectomy

In the univariate logistic regression analysis, male sex, smoking, an ASA score ≥3, neoadjuvant chemotherapy, stage III or IV cancer, total gastrectomy, open surgery, D2 or more lymph node dissection, and longer operation times were significantly associated with SSI. After checking the multicollinearity, gender, smoking history, surgical method, extent of surgery, and staging, and one of the body composition criteria were selected for multivariate logistic regression analysis based on the clinical significance. The results of the logistic regression analysis, including the VFA-to-SFA ratio, are shown in Table 2. A multivariate logistic analysis including the interaction term indicated that total gastrectomy (OR, 2.13; *p* = 0.017), stage III or IV cancer (OR, 2.66; *p* = 0.003), and a high VFA-to-SFA ratio (OR, 8.09; *p* < 0.001) were independent risk factors for SSI after gastrectomy. The effects of the interacting variables are presented separately in Supplementary Table S1.

### 3.3. Comparison of Cody Composition Variables to Predict SSI after Gastrectomy

Using the ROC curve and the AUC value, the body composition criteria variable with the highest predictive power for SSI was determined. As shown in Table 3, the AUC value of Model 1-6, which was subjected to multivariate logistic regression analysis by adding the VFA-to-SFA ratio to the other existing variables, was 75.11, which is higher than the AUC of the other models. In addition, the Hosmer–Lemeshow test was performed to evaluate the predictive performance of the model. The model was identified as appropriate, with a *p*-value of 0.3812. The superiority of the VFA-to-SFA ratio to the control was identified using the DeLong method using a *p*-value of 0.022. These results were also applied to the organ/space SSI, and the results about organ/space SSI are shown in Supplementary Tables.

### 3.4. The Threshold Values of the VFA-To-SFA Ratios are related to SSI after Gastrectomy 

**Figure 3:**
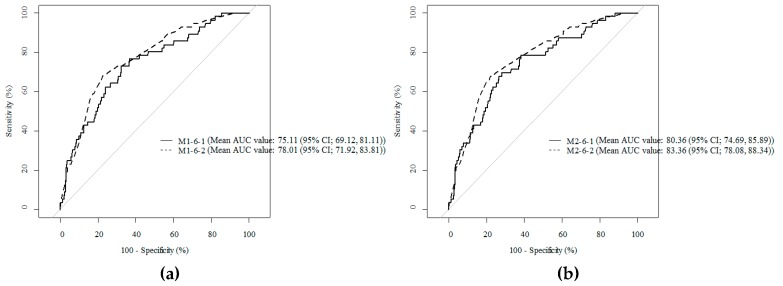
Receiver operating characteristic curve to compare performance of models to predict surgical site infection after gastrectomy by using 1000 bootstrap samples (**a**: all surgical site infections; M1-6-1: visceral fat area to subcutaneous fat area as a continuous variable; M1-6-2: visceral fat area to subcutaneous fat area as a binary variable with cut-off point 0.94) (**b**: organ/space surgical site infection; M2-6-1: visceral fat area to subcutaneous fat area as a continuous variable; M2-6-2: visceral fat area to subcutaneous fat area as a binary variable with a cut-off point of 0.94).

The cutoff point of the VFA-to-SFA ratio was 0.94, using the Youden method to improve the predictive power of the SSI. To compare the performance of Model M1-6-1 using the VFA-to-SFA ratio as a continuous variable, and Model M1-6-2 using the VFA-to-SFA ratio as a binary variable with a cutoff point of 0.94, we used 1000 bootstrap samples, and the mean AUC value was obtained and the results are shown in Figure 3. In addition, the sensitivity, specificity, positive predictive value (PPV), negative predictive value (NPV), accuracy, and AUC value calculated when differentiating the VFA-to-SFA using the cutoff value of 0.94 are shown in Table 4.

### 3.5. Comparison of the Treatment Outcomes after Gastrectomy according to Abdominal Fat Composition

Table 5 shows the differences in the treatment outcomes after gastrectomy depending on the composition of abdominal fat. In the high VFA-to-SFA ratio group, the SSI occurrence was statistically significantly higher than in the low VFA-to-SFA ratio group (8.4% vs. 1.4%, respectively; *p* < 0.001). This was mainly due to the organ/space SSI (7.1% vs. 0.5%, respectively; *p* < 0.001). In the high VFA-to-SFA ratio group, the incidence rate of a Clavien-Dindo score of IIIa or a higher for postoperative complication, mean days of postoperative hospital stay, and the incidence rate of re-admission within 30 days were significantly higher than in the low VFA-to-SFA ratio group. However, there was no difference in the mortality rate between the two groups (0.3% vs. 0.2%, respectively, *p* = 1.000). 

## 4. Discussion

This study demonstrated that the VFA-to-SFA ratio had the highest predictive power for SSI after gastrectomy among the body composition criteria assessed, especially the organ/space SSI. Moreover, the VFA-to-SFA ratio could be widely applicable, because it is a value defined by a ratio, not an absolute value. The large number of patients who underwent gastrectomy in our study increased the clinical significance of this result. 

A high VFA-to-SFA ratio indicates either an increased VFA or a decreased SFA. As the area of the abdominal fat and muscle is obtained through the CT scan, several studies have shown that VFA is better than BMI at predicting postoperative complications, including SSI. One reason patients with visceral obesity are more likely to develop an SSI could be due to the surgical difficulty associated with the surgeon having a deeper and poorer view of the surgical field, as well as the fragile, easily bleeding tissue in high-visceral obesity patients [7]. In addition, visceral obesity is expected to increase post-operative morbidity, because visceral adipose tissue secretes a number of adipokines and cytokines leading to a proinflammatory, procoagulant, and an insulin-resistant state, collectively known as the metabolic syndrome [27,28]. Thus, patients with visceral obesity often have higher ASA scores or more comorbidities, including diabetes and cardiovascular diseases [6]. These are the reasons SSI, especially organ/space SSI, occurs more frequently in patients with visceral obesity. Related to these findings, Takeuchi et al. classified VFA into two groups based on a cutoff of 100 cm^2^, and reported that SSI occurred more frequently in the high VFA group [8]. However, in our study, when compared using the ROC curve and AUC value, the VFA-to-SFA ratio was more predictive for the SSI than the VFA itself. 

Conversely, SFA improves insulin sensitivity. Thus, it can work as a buffer against the lipid accumulation of VFA. Consequently, SFA insufficiency causes increased lipid accumulation in visceral fat, and therefore the VFA-to-SFA ratio is effective for evaluating the risk of diseases affected by the adipose tissue [29]. For this reason, several studies on the influence of the VFA-to-SFA ratio have been conducted in various fields in recent years. Yosuke et al. reported that the VFA-to-SFA ratio was an independent risk factor for decreased renal function in kidney transplantation recipients [30]. Kaess et al. reported that the VFA-to-SFA ratio is correlated with cardiometabolic risk, more than BMI and VFA itself [31]. Our study revealed that these findings can also be applied to the risk prediction of SSI after gastrectomy. Traditionally, in addition to visceral obesity, sarcopenia is known to be a major risk factor for SSI after gastrectomy. Tatsuko et al. reported that sarcopenic obesity, which was defined as a skeletal muscle mass index of ≤52.4 cm^2^/m^2^ for men and ≤38.5 cm^2^/m^2^ for women, and a visceral fat area of ≥100 cm^2^ in both sexes, is an independent risk factor for the development of SSI after laparoscopic total gastrectomy [32]. However, the VFA-to-SFA ratio was more predictive for SSI than sarcopenia. Also, the above study defined sarcopenia and obesity based on specific values, which would be difficult to apply widely.

Recently, some studies have been published that address the risk of postoperative complications, such as SSI, by values calculated by a ratio rather than a specific value based on body composition criteria. According to Pecorelli et al., the high VFA-to-TAMA ratio was a major risk factor for postoperative complications, especially in pancreatic cancer patients [33,34]. However, in our study using a ROC curve, the AUC value of the VFA-to-SFA ratio was greater than that of the VFA-to-TAMA ratio, making the VFA-to-SFA ratio a better predictor of SSI than the VFA-to-TAMA ratio after gastrectomy.

In addition to the individual fat distribution, total gastrectomy and stage III or IV cancer also influenced the occurrence of SSI. Total gastrectomy is one of the most invasive gastrointestinal surgeries, and the risk of SSI after a total gastrectomy is known to be higher than after a partial gastrectomy [35,36]. Thus, this finding is consistent with the conclusions of previous studies. On the other hand, male sex, smoking, and open surgery, which were also known as risk factors of SSI, did not show statistically significant associations in multivariate analysis after adjusting the effect of the VFA-to-SFA ratio. A relatively small number of events might have limited the statistical power to detect associations. If the sample size gets larger, these variables may also be statistically significant, especially the male gender with a high OR and wide confidence interval.

This study had several limitations. First, this study was a nonrandomized retrospective study of a single country with a homogenous ethnic background. Second, we excluded 10 patients with follow-up loss or incomplete data review of the 1067 patients who underwent a gastrectomy in 2015. Although these patients did not develop SSI during the hospitalization, and the rate of SSI occurrence after discharge was low (12/1038; 1.2%), we could not completely rule out the possibility that SSI occurred after discharge in these patients. Third, active post-discharge surveillance using phone calls or family physicians has not been conducted. Thus, there was a potential for some SSI incidents to be missed. Fourth, as a result of the model comparison, the 95% confidence interval of the AUC value of each model overlapped. Fifth, in the present study, the incidence of SSI after gastrectomy was 5.6%, which is not high. Therefore, this raises the issue that the sensitivity and PPV were low for predicting the increased risk of SSI with a specific cutoff value. However, SSI is a complication that cannot be accurately predicted before surgery, so it is meaningful to find patients who are at high risk for SSI before surgery. Surgeons may consider minimal invasive surgical methods and try to reduce the operation time so as to reduce SSI in patients with a high VFA-to-SFA ratio. In this regard, even though the sensitivity and PPV were low, the specific cutoff value we determined would be beneficial. In addition, the high NPV of the VFA-to-SFA ratio cutoff value allows surgeons to identify patients at a very low risk of developing SSI before surgery. Further research is needed to determine if it will be applicable in other abdominal surgeries and other countries.

## 5. Conclusions

A high VFA-to-SFA ratio is an independent risk factor for the development of SSI after gastrectomy. Also, the results of this study suggest that the VFA-to-SFA ratio is the most predictable body composition model to predict the occurrence of SSI after gastrectomy. It would be beneficial for surgeons to pay greater attention to SSI when operating on patients with high VFA-to-SFA ratios.

## Figures and Tables

**Table 1:** Clinical characteristics of patients with surgical site infection after gastrectomy.

	Total (n = 1038)	SSI (−) (n = 980)	SSI (+) (n = 58)	*p*-Value
Age	59 (50–67)	59 (50–67)	62.5 (53–68)	0.206
Sex				0.037
Male	654 (63)	610 (62.2)	44 (75.9)	
Female	384 (37)	370 (37.8)	14 (24.1)	
BMI >25	330 (31.8)	308 (31.4)	22 (37.9)	0.301
Smoking	561 (54)	520 (53.1)	41 (70.7)	0.009
ASA score				0.142
>3	794 (76.5)	756 (77.1)	38 (65.5)	
≤3	244 (23.5)	141 (22.9)	11 (34.5)	
Hypertension	338 (32.6)	313 (31.9)	25 (43.1)	0.078
Diabetes	150 (14.5)	140 (14.3)	10 (17.2)	0.534
Cerebrovascular accident	32 (3.1)	31 (3.2)	1 (1.7)	1.000
Arrhythmia	19 (1.8)	19 (1.9)	0 (0)	0.621
Congestive heart failure	16 (1.5)	16 (1.6)	0 (0)	1.000
Coronary artery disease	43 (4.1)	41 (4.2)	2 (3.4)	1.000
Chronic pulmonary disease	14 (1.3)	14 (1.4)	0 (0)	1.000
Old tuberculosis	79 (7.6)	72 (7.3)	7 (12.1)	0.188
Chronic kidney disease	48 (4.6)	44 (4.5)	4 (6.9)	0.337
Neoadjuvant chemotherapy	53 (5.1)	46 (4.7)	7 (12.1)	0.013
Pathological stage				<0.001
Stage I and II	800 (79.3)	768 (80.6)	32 (57.1)	
Stage III and IV	209 (20.7)	185 (19.4)	24 (42.9)	
Extent of surgery				<0.001
Total gastrectomy	228 (22)	203 (20.7)	25 (43.1)	
Partial gastrectomy	810 (78)	777 (79.3)	33 (56.9)	
Surgical approach				0.005
Open	441 (42.5)	406 (41.4)	35 (60.3)	
Minimally invasive	597 (57.5)	574 (58.6)	23 (39.7)	
Combined resection	105 (10.1)	97 (9.9)	8 (13.8)	0.339
Lymph node dissection				0.012
D1 and D1+	451 (43.4)	435 (44.4)	16 (27.6)	
D2 or more	587 (56.6)	545 (55.6)	42 (72.4)	
Operation time (min)	177 (145–211)	175 (144–210)	203 (171.5–242)	<0.001

Data are presented as median (interquartile range) or number (%) of patients, unless otherwise indicated. SSI: surgical site infection; BMI: body mass index; ASA: American Society of Anesthesiologists.

**Table 2:** Multivariate logistic regression analysis * to identify independent risk factors for surgical site infection after gastrectomy.

	OR (95% CI)	*p*-Value
Gender		
Female	Ref	
Male	3.71 (0.79, 17.42)	0.097
Smoking		
No	Ref	
Yes	1.79 (0.74, 4.34)	0.196
Surgical method		
Minimal invasive	Ref	
Open surgery	1.12 (0.58, 2.16)	0.737
Extent of surgery		
Partial resection	Ref	
Total resection	2.13 (1.15, 3.94)	0.017
Staging		
Stage I or II	Ref	
Stage I or IV	2.66 (1.39, 5.08)	0.003
VFA-to-SFA ratio	8.09 (2.91, 22.54)	<0.001

OR: odds ratio; CI: confidence interval; VFA: visceral fat area; SFA: subcutaneous fat area; Ref: reference * Analysis including interaction term (estimated interaction OR, 0.19; 95% CI, 0.06–0.55; *p*-value 0.002).

**Table 3:** Comparison of body composition variables to predict surgical site infection after gastrectomy using receiver operating characteristic analyses.

Model	Added Variable	Mean AUC (95% CI)	*p*-Value *
M1-1	Control	71.38(64.34, 78.57)	0.447
M1-2	VFA	73.84(66.84, 80.38)	0.700
M1-3	TAMA	71.81(64.76, 78.86)	0.466
M1-4	Muscle index	71.65(64.80, 78.88)	0.591
M1-5	Visceral fat index	73.41(66.48, 79.95)	0.679
M1-6	VFA-to-SFA	75.11(69.12, 81.11)	0.381
M1-7	VFA-to-TAMA	73.47(66.37, 79.90)	0.786
M1-8	VFA-to-Muscle index	73.97(66.91, 80.30)	0.812

AUC: area under the curve; CI: confidence interval; VFA: visceral fat area; TAMA: total abdominal muscle area; SFA: subcutaneous fat area. * evaluated by the Hosmer–Lemeshow test.

**Table 4:** Diagnostic performance of model to predict surgical site infection after gastrectomy using visceral fat area to subcutaneous fat area as a binary variable.

	For Model (Using the Binary Variable)
VFA-to-SFA cut-off value	0.94
Sensitivity (95% CI) %	67.86 (55.63, 80.09)
Specificity (95% CI) %	77.65 (75.00, 80.29)
PPV (95% CI) %	15.14 (10.71, 19.57)
NPV (95% CI) %	97.63 (96.54, 98.71)
Accuracy (95% CI) %	77.11 (74.51, 79.70)
AUC (95% CI) %	72.75 (66.44, 79.06)

VFA: visceral fat area; SFA: subcutaneous fat area; CI: confidence interval; PPV: positive predictive value; NPV: negative predictive value; AUC: area under the curve.

**Table 5:** Comparison of treatment outcomes after gastrectomy according to abdominal fat composition.

	Total (n = 1038)	VFA-to-SFA ≥0.94 (n = 616)	VFA-to-SFA <0.94 (n = 422)	*p*-Value
SSI occurrence	58 (5.6)	52 (8.4)	6 (1.4)	<0.001
Type of SSI				
Incisional	12 (1.2)	8 (1.3)	4 (0.9)	0.771
Organ/space	46 (4.4)	44 (7.1)	2 (0.5)	<0.001
Clavien-Dindo score of IIIa or higher postoperative complication	78 (7.5)	59 (9.6)	19 (4.5)	0.002
Postoperative hospital stay (days)	7.64 ± 5.72	8.1 ± 6.96	6.95 ± 3.01	<0.001
Re-admission within 30 days	42 (4)	33 (5.4)	9 (2.1)	0.011
Mortality	3 (0.3)	2 (0.3)	1 (0.2)	1.000

Data are presented as mean ± standard deviation or number (%) of patients, unless otherwise indicated. VFA: visceral fat area; SFA: subcutaneous fat area; SSI: surgical site infection.

## Supplementary Materials

The following are available online at https://www.mdpi.com/2077-0383/8/4/494/s1, Table S1: Subgroup analysis by gender to identify independent risk factors for surgical site infection after gastrectomy using multivariate logistic regression analysis *, Table S2: Comparison of body composition variables to predict organ/space surgical site infection after gastrectomy using receiver operating characteristic analyses, Table S3: Diagnostic performance of model to predict organ/space surgical site infection after gastrectomy using visceral fat area to subcutaneous fat area as a binary variable.
