# Supplementary material for: A High Visceral-To-Subcutaneous Fat Ratio is an Independent Predictor of Surgical Site Infection after Gastrectomy
Source: J Clin Med. 2019 Apr 11;8(4):494. doi: 10.3390/jcm8040494 (PMC6518224; doi:10.3390/jcm8040494)
Supplement: Supplementary file 1 [file jcm-08-00494-s001.pdf]

Table S1. Subgroup analysis by gender to identify independent risk factors for surgical site infection after gastrectomy using multivariate logistic regression analysis *.

	Female		Male	
	**OR (95% CI)**	**p-value**	**OR (95% CI)**	**p-value**
VFA-to-SFA ratio	9.78 (3.37, 28.41)	<0.001	1.48 (1.02, 2.16)	0.039

OR: odds ratio; CI: confidence interval; VFA: visceral fat area; SFA: subcutaneous fat area. * Adjusted for smoking, surgical method, extent of surgery, staging.

Table S2. Comparison of body composition variables to predict organ/space surgical site infection after gastrectomy using receiver operating characteristic analyses.

Model	Added variable	Mean AUC (95% CI)	p-value*
**M2-1**	Control	75.61(68.17, 82.28)	0.405
**M2-2**	VFA	79.39(72.71, 85.27)	0.295
**M2-3**	TAMA	76.34(69.18, 82.97)	0.636
**M2-4**	Muscle index	75.97(68.44, 82.63)	0.064
**M2-5**	Visceral fat index	78.95(72.31, 84.91)	0.921
**M2-6**	VFA-to-SFA	80.36(74.69, 85.89)	0.567
**M2-7**	VFA-to-TAMA	78.55(72.17, 84.62)	0.583
**M2-8**	VFA-to-Muscle index	79.01(72.25, 85.15)	0.436

AUC: area under the curve; CI: confidence interval; VFA: visceral fat area; TAMA: total abdominal muscle area; SFA: subcutaneous fat area. * evaluated by Hosmer-Lemeshow test.

Table S3. Diagnostic performance of model to predict organ/space surgical site infection after gastrectomy using visceral fat area to subcutaneous fat area as a binary variable.

	For model (using the binary variable)
VFA-to-SFA cut-off value	0.94
Sensitivity(95% CI) %	75.00(62.21, 87.79)
Specificity(95% CI) %	77.62(74.99, 80.25)
PPV(95% CI) %	13.25(9.04, 17.46)
NPV(95% CI) %	98.55(97.70, 99.40)
Accuracy(95% CI) %	77.50(74.93, 80.08)
AUC(95% CI) %	76.31(69.70, 82.91)

SSI: surgical site infection; VFA: visceral fat area; SFA: subcutaneous fat area; CI: confidence interval; PPV: positive predictive value; NPV: negative predictive value; AUC: area under the curve.
